# Breastfeeding and myopia: A cross-sectional study of children aged 6–12 years in Tianjin, China

**DOI:** 10.1038/s41598-018-27878-0

**Published:** 2018-07-03

**Authors:** Shengxin Liu, Sheng Ye, Qifan Wang, Yongjun Cao, Xin Zhang

**Affiliations:** 1 Department of Maternal, Child and Adolescent Health, School of Public Health, Tianjin Medical University, No. 22 Qixiangtai Road, Tianjin, 300070 China; 2 Department of Obstetrics and Gynecology, School of Nursing, Tianjin Medical University, No. 22 Qixiangtai Road, Tianjin, 300070 China

## Abstract

This study aimed to determine whether an association existed between breastfeeding and myopia in children aged 6–12 years in Tianjin, China, using a cross-sectional study of 527 children. The spherical equivalent refraction (SER) and axial length (AL) were determined by ocular examination, and information regarding the children’s demographics, breastfeeding history and other myopia-related risk factors were investigated using a questionnaire. The myopia prevalence rate, mean SER, and mean AL were 53.9%, −0.99 ± 1.69 D, and 23.56 ± 1.11 mm, respectively. In total, 442 (83.9%) participants were breastfed; among the breastfed participants, 132 (29.9%) were breastfed <6 months. Children who were breastfed were less likely to have myopia (breastfeeding duration <6 months: OR = 0.399, *P* = 0.011; >6 months: OR = 0.502, *P* = 0.033, multiple logistical regression). The mean SER of children breastfed <6 months was 0.653 D more hyperopic than that of non-breastfed children (*P* = 0.008, multiple linear regression). No significant association was observed between breastfeeding and AL. In conclusion, breastfeeding was associated with a decreased risk of myopia among children aged 6–12 years in Tianjin. Breastfeeding during the first 6 months of infancy was associated with more hyperopic SER. Furthermore, breastfeeding was associated with myopic refraction and was not related to AL, and this association could exist in childhood.

## Introduction

Myopia is one of the most common types of visual impairment^1,2^. Myopia not only affects students’ education and quality of life but also impairs their physical and mental health^3^. In recent decades, myopia has become a significant worldwide public health concern due to its increasing prevalence^4,5^. An estimated 2.62 billion people will suffer from myopia by the year 2020, and approximately 4.76 billion people (49.8% of the global population) will become myopic by the end of 2050^6^. Epidemiological studies have indicated that the prevalence rates of myopia in Asian countries, including China, Korea and Singapore, are higher than those in Europe, Australia and other countries^7–12^. Notably, in several schools in Beijing, China, the prevalence rate of myopia in children aged 7–18 years old was 64.9%^8^. In Korea, the prevalence rates of myopia in children aged 5–6, 7–11, and 12–18 years were 20.4%, 58.4% and 80.2%, respectively, from 2008–2012^9^, whereas the myopia prevalence was relatively lower in European countries. For example, the myopia prevalence rate in 12-13-year-old students was only 17.7% in Northern Ireland^11^.

Over the past few decades, extensive studies have been conducted to explore the risk factors of myopia^13,14^. Although the explanatory factors for myopia are currently uncertain, studies have suggested that environmental factors play a critical role. Furthermore, nutrition intake, particularly breastfeeding during the infantile period, is considered to influence visual development and eye growth^15^. A prospective study has revealed that children aged 3.5 years who are initially breastfed have better vision status than those who are fed by formula milk^16^. Additionally, a Singapore cohort study of 797 subjects aged 10–12 years suggested that breastfeeding was a protective factor for myopia after adjusting for age, gender, parental myopia, IQ score and so on^17^. However, other studies have reported inconsistent results. One study conducted in 2008 to investigate the association between infant feeding and visual outcome demonstrated that in 3 British birth cohorts, breastfeeding did not appear to influence visual development after adjustment for age^18^. Another cross-sectional study conducted in Iran that included 367 children ranging in age from 6–10 years showed that breastfeeding was not significantly associated with refractive errors^19^. This discrepancy among studies may be caused by differences in the nature of the studies and participants age and ethnicity, as well as adjustments for different confounders, etc. Furthermore, Sham *et al*.^20^ showed that the effects of breastfeeding might be concealed by ocular development at ages from 6–72 months and suggested that breastfeeding might have a marked impact on myopia in older children. Chong *et al*.^17^ also showed that the effect of breastfeeding on myopia could persists later into childhood. Thus, the relationship between breastfeeding and myopic refraction in the Chinese population remains uncertain, and it is rarely known whether this relationship could exist in childhood.

Previous studies have mostly focused on the relationship between breastfeeding and refraction without considering ocular biometric parameters. Studying the effects of breastfeeding on ocular biometric parameters can help clarify the specific mechanism underlying the association between breast milk and myopia. The axial length (AL) of the eye is the main determinant of eyeball size and a basic ocular biometric parameter. Since the central part of the retina provides the greatest vision acuity, AL has substantial importance for refraction^21^. Wickremasinghe *et al*.^22^ revealed that AL was the strongest determinant of the refractive status in multiple regression models. Another study also suggested that longer ALs were significantly associated with myopia^23^. Thus, it is very necessary to evaluate the association between breastfeeding and AL.

To the best of our knowledge, studies detailing the relationship between breastfeeding and myopic refraction in China are rare. Moreover, whether breastfeeding is associated with myopic refraction and whether this association exists in childhood remain controversial. The impacts of breastfeeding on ocular biometric parameters, such as the AL, deserves further study to illustrate the mechanism underlying the association between breastfeeding and myopia. Thus, this study aimed to examine the association between breastfeeding and myopic refraction in children aged 6–12 years in China accounting for the potential confounding factors, to investigate the prevalence of myopia in Tianjin, China, and to evaluate whether breastfeeding is associated with AL. We hoped that our findings will help explain the impacts of early nutritional intake, such as breast milk, on myopia and eye development and provide clues to the aetiology of myopia in Chinese school-aged children.

## Results

### Characteristics of the participants

A total of 527 children aged 6–12 years were included in this study (285 boys (54.1%) and 242 girls (45.9%)). The mean age of the participants was 9.13 ± 1.73 years, and the overall prevalence rate of myopia was 53.9% (284 of 527; 95% CI: 49.6–58.2%). The mean spherical equivalent refraction (SER) was −0.99 ± 1.69 D, and the mean AL was 23.56 ± 1.11 mm. Among the study participants, 442 participants (83.9%) were breastfed, including 132 (29.9%) who were breastfed less than 6 months, and 118 (26.7%) who were breastfed longer than 12 months.

### Univariate analysis of myopia prevalence

As shown in Table 1, age was associated with myopia (*P* < 0.001). Children who had one or two myopic parents were more likely to be myopic than were those without myopic parents (*P* = 0.024). The prevalence rate of myopia was significantly lower in children who were breastfed (51.8%) than in non-breastfed children (64.7%) (*P* = 0.029). In addition, the duration of breastfeeding (never, <6 months, 6–12 months, and >12 months) was associated with the prevalence of myopia (*P* = 0.043).

**Table 1:** Characteristics of the study participants and myopia prevalence (univariate).

Characteristics	Participants	With myopia	Without myopia	*P*-value
**Age, mean (±SD), years**	527	9.66 (±1.72)	8.51 (±1.54)	<0.001**
**Gender, n (%)**				0.342
Boys	285	159 (55.8)	126 (44.2)	
Girls	242	125 (51.7)	117 (48.3)	
**Birth height, mean (±SD), cm**	442	51.45 (±4.55)	50.92 (±4.04)	0.198
**Birth weight, mean (±SD), 500 g**	465	6.81 (±1.17)	6.65 (±1.04)	0.122
**Maternal educational level, n (%)**				0.645
Senior high school and lower	91	49 (53.8)	42 (46.2)	
College	143	75 (52.4)	68 (47.6)	
University	215	122 (56.7)	93 (43.3)	
Master’s and higher	78	38 (48.7)	40 (51.3)	
**Parental myopia, n (%)**				0.024*
None	133	62 (46.6)	71 (53.4)	
One	226	118 (52.2)	108 (47.8)	
Both	168	104 (61.9)	64 (38.1)	
**Monthly household income, n (%)**				0.211
<4000 RMB	49	29 (59.2)	20 (40.8)	
4000–8000 RMB	145	74 (51.0)	71 (49.0)	
8000–10,000 RMB	149	71 (47.7)	78 (52.3)	
10,000–15,000 RMB	103	63 (61.2)	40 (38.8)	
>15,000 RMB	79	45 (57.0)	34 (43.0)	
**Maternal age, mean (±SD), years**	511	27.79 (±3.05)	28.00 (±3.35)	0.459
**Mode of delivery, n (%)**				0.841
Vaginal delivery	169	90 (53.3)	79 (46.7)	
Caesarean delivery	358	194 (54.2)	164 (45.8)	
**Gestational weeks, n (%)**				0.691
Postmature delivery (>42 weeks)	51	26 (51.0)	25 (49.0)	
Term delivery (37–41 weeks)	427	229 (53.6)	198 (46.4)	
Premature delivery (<36 weeks)	49	29 (59.2)	20 (40.8)	
**Maternal smoking history during pregnancy, n (%)**				0.912
Yes	2	1 (50.0)	1 (50.0)	
No	525	283 (53.9)	242 (46.1)	
**Maternal passive smoking history during pregnancy, n (%)**				0.401
Yes	265	138 (52.1)	127 (47.9)	
No	262	146 (55.7)	116 (44.3)	
**Maternal drinking history during pregnancy, n (%)**				0.033*
Yes	40	28 (70.0)	12 (30.0)	
No	487	256 (52.6)	231 (47.4)	
**Outdoor time, mean (±SD), h/day**	527	1.23 (±0.79)	1.36 (±0.97)	0.092
**Near work time, mean (±SD), h/day**	527	6.32 (±3.37)	5.26 (±3.16)	0.043*
**Breastfed, n (%)**				0.029*
Yes	442	229 (51.8)	213 (48.2)	
No	85	55 (64.7)	30 (35.3)	
**Duration of breastfeeding, n (%)**				0.043*
Never	85	55 (64.7)	30 (35.3)	
<6 months	132	64 (48.5)	68 (51.5)	
6–12 months	192	109 (56.8)	83 (43.2)	
>12 months	118	56 (47.5)	62 (52.5)	
**SER (right eye), mean (±SD), D**	527	−2.13 (±1.39)	0.35 (±0.76)	<0.001**
**AL (right eye), mean (±SD), mm**	527	24.06 (±1.11)	22.97 (±0.76)	<0.001**

SD, standard deviation. SER, spherical equivalent refraction. *P < 0.05; **P < 0.01.

### Multivariate logistical regression analysis of the association between breastfeeding and myopia

The relationship between breastfeeding duration and myopic status is shown in Table 2. After adjusting for covariates, the OR for myopia in children breastfed for less than 6 months was 0.399 (95% CI: 0.196–0.811, *P* = 0.011) compared with non-breastfed children. Children who were breastfed more than 6 months had a 49.8% lower risk of having myopia than did those who were not breastfed (95% CI: 0.266–0.946, *P* = 0.033). Furthermore, children with two myopic parents had the highest risk of myopia (OR = 2.389, 95% CI: 1.269–4.499, *P* = 0.007). Finally, children with mothers who had no drinking history during pregnancy were less likely to have myopia than were children with mothers with a maternal history of drinking during pregnancy (OR = 0.386, 95% CI: 0.154–0.964, *P* = 0.042).

**Table 2:** Factors associated with the prevalence of myopia (multivariable).

Characteristics	OR	95% CI	OR (95% CI)	*P*-value
**Age**	1.519	1.311, 1.759		<0.001**
**Gender**				
Boys	1.000			
Girls	0.920	0.597, 1.418		0.706
**Birth height**	1.014	0.962, 1.068		0.609
**Birth weight**	1.249	0.996, 1.567		0.055
**Maternal educational level**				
Senior high school and lower	1.000			
College	0.726	0.357, 1.476		0.377
University	0.705	0.341, 1.458		0.346
Master’s and higher	0.518	0.204, 1.317		0.167
**Parental myopia**				
None	1.000			
One	1.295	0.740, 2.266		0.365
Both	2.389	1.269, 4.499		0.007**
**Monthly household income**				
<4000 RMB	1.000			
4000-8000 RMB	1.106	0.451, 2.641		0.823
8000-10,000 RMB	0.885	0.364, 2.193		0.791
10,000-15,000 RMB	1.875	0.737, 4.981		0.199
>15,000 RMB	1.396	0.513, 3.978		0.525
**Maternal reproductive age**	1.007	0.939, 1.080		0.849
**Mode of delivery**				
Vaginal delivery	1.000			
Caesarean delivery	0.640	0.399, 1.028		0.065
**Gestational weeks**				
Postmature delivery (>42 weeks)	1.000			
Term delivery (37-41 weeks)	1.320	0.631, 2.762		0.460
Premature delivery (<36 weeks and less)	1.851	0.510, 5.615		0.277
**Maternal smoking history during pregnancy**				
Yes	1.000			
No	0.000	0.000, ----		1.000
**Maternal passive smoking history during pregnancy**				
Yes	1.000			
No	1.098	0.710, 1.697		0.675
**Maternal drinking history during pregnancy**				
Yes	1.000			
No	0.386	0.154, 0.964		0.042*
**Outdoor time**	0.877	0.669, 1.150		0.341
**Near work time**	1.039	0.970, 1.114		0.272
**Duration of breastfeeding**				
Never	1.000			
<6 months	0.399	0.196, 0.811		0.011*
>6 months	0.502	0.266, 0.946		0.033*

CI, confidence interval; OR, odds ratio. *P < 0.05; **P < 0.01.

### Multivariate linear regression analysis of the association between breastfeeding and SER

The association between SER and breastfeeding is shown in Table 3. After adjusting for the same factors, the mean SER of children who were breastfed less than 6 months was 0.653 D more hyperopic than that of children who were not breastfed (*P* = 0.008). In addition, the difference in the mean SER values between children who were breastfed more than 6 months (0.346 D, *P* = 0.117) and non-breastfed children was not significant. The mean SER decreased by 0.383 D (*P* < 0.001) for each increase in age of 1 year when controlling for other risk factors. The mean SER of children with mothers holding university degrees was increased by 0.667 D (*P* = 0.009) compared with that of children from mothers with senior high school and lower educational levels. Similarly, children with mothers holding Master’s and higher degrees had significantly more hyperopic refraction (*B* = 0.951, *P* = 0.004) than those with mothers with senior high school and lower educational levels. The mean SERs in children with two or one myopic parent were significantly more myopic by 0.982 D (*P* < 0.001) and 0.454 D (*P* = 0.022), respectively, versus the mean SERs in children with no myopic parents.

**Table 3:** Factors associated with the SER (multivariable).

Characteristics	Regression coefficient B	SE	95% CI	*P*-value
**Age**	−0.383	0.048	−0.478, −0.287	<0.001**
**Gender**				
Boys	0 (reference)			
Girls	0.145	0.152	−0.155, 0.444	0.343
**Birth height**	0.007	0.018	−0.028, 0.042	0.690
**Birth weight**	−0.109	0.078	−0.262, 0.044	0.163
**Maternal educational level**				
Senior high school and lower	0 (reference)			
College	0.356	0.251	−0.137, 0.849	0.157
University	0.667	0.256	0.165, 1.170	0.009**
Master’s and higher	0.951	0.329	0.304, 1.599	0.004**
**Parental myopia**				
None	0 (reference)			
One	−0.454	0.197	−0.842, −0.066	0.022*
Both	−0.982	0.217	−1.410, −0.555	<0.001**
**Monthly household income**				
<4000 RMB	0 (reference)			
4000–8000 RMB	0.028	0.312	−0.586, 0.642	0.929
8000–10,000 RMB	−0.094	0.319	−0.721, 0.533	0.769
10,000–15,000 RMB	−0.790	0.339	−1.456, −0.124	0.020*
>15,000 RMB	−0.463	0.360	−1.170, 0.244	0.198
**Maternal reproductive age**	−0.029	0.025	−0.078, 0.019	0.236
**Mode of delivery**				
Vaginal delivery	0 (reference)			
Caesarean delivery	0.070	0.164	−0.252, 0.393	0.668
**Gestational weeks**				
Postmature delivery (>42 weeks)	0 (reference)			
Term delivery (37–41 weeks)	−0.029	0.262	−0.543, 0.485	0.912
Premature delivery (<36 weeks)	−0.158	0.388	−0.920, 0.604	0.684
**Maternal smoking history during pregnancy**				
Yes	0 (reference)			
No	1.428	1.560	−1.639, 4.495	0.361
**Maternal passive smoking history during pregnancy**				
Yes	0 (reference)			
No	−0.263	0.153	−0.564, 0.038	0.087
**Maternal drinking history during pregnancy**				
Yes	0 (reference)			
No	0.232	0.304	−0.365, 0.829	0.446
**Outdoor time**	−0.033	0.095	−0.218, 0.152	0.726
**Near work time**	−0.040	0.024	−0.087, 0.008	0.102
**Duration of breastfeeding**				
Never	0 (reference)			
<6 months	0.653	0.246	0.170, 1.136	0.008**
>6 months	0.346	0.220	−0.086, 0.778	0.117

SE, standard error; CI, confidence interval; *P < 0.05; **P < 0.01.

### Multivariate linear regression analysis of the association between breastfeeding and the AL

No significant association was found between AL and breastfeeding after adjusting for the effect of the other related risk factors (Table 4). In addition, in the multiple linear regression model, longer ALs were associated with an older age (*P* < 0.001), boys (*P* < 0.001), a greater body weight (*P* < 0.001), one myopic parent (*P* = 0.022), two myopic parents (*P* = 0.004), a monthly household income of 10,000–15,000 RMB (*P* = 0.018), no maternal passive smoking history during pregnancy (*P* = 0.004) and more time spent near work (*P* = 0.018).

**Table 4:** Factors associated with AL (multivariable).

Characteristics	Regression coefficient B	SE	95% CI	*P*-value
**Age**	0.301	0.028	0.246, 0.356	<0.001**
**Gender**				
Boys	0 (reference)			
Girls	−0.597	0.088	−0.770, −0.424	<0.001**
**Birth height**	0.004	0.010	−0.017, 0.024	0.723
**Birth weight**	0.162	0.045	0.074, 0.251	<0.001**
**Maternal educational level**				
Senior high school and lower	0 (reference)			
College	0.018	0.145	−0.267, 0.304	0.899
University	−0.212	0.148	−0.502, 0.079	0.153
Master’s and higher	−0.209	0.190	−0.583, 0.166	0.274
**Parental myopia**				
None	0 (reference)			
One	0.262	0.114	0.037, 0.486	0.022*
Both	0.367	0.126	0.120, 0.614	0.004 **
**Monthly household income**				
<4000 RMB	0 (reference)			
4000–8000 RMB	0.022	0.181	−0.333, 0.377	0.904
8000–10,000 RMB	0.086	0.184	−0.276, 0.449	0.640
10,000–15,000 RMB	0.466	0.196	0.081, 0.851	0.018*
>15,000 RMB	0.120	0.208	−0.289, 0.529	0.564
**Maternal reproductive age**	0.011	0.014	−0.017, 0.040	0.424
**Mode of delivery**				
Vaginal delivery	0 (reference)			
Caesarean delivery	−0.106	0.095	−0.292, 0.081	0.267
**Gestational weeks**				
Postmature delivery (>42 weeks)	0 (reference)			
Term delivery (37–41 weeks)	−0.001	0.151	−0.298, 0.297	0.996
Premature delivery (<36 weeks)	0.103	0.224	−0.338, 0.544	0.646
**Maternal smoking history during pregnancy**				
Yes	0 (reference)			
No	−0.471	0.902	−2.244, 1.303	0.602
**Maternal passive smoking history during pregnancy**				
Yes	0 (reference)			
No	0.255	0.089	0.080, 0.429	0.004**
**Maternal drinking history during pregnancy**				
Yes	0 (reference)			
No	−0.165	0.176	−0.510, 0.180	0.348
**Outdoor time**	−0.032	0.054	0.561, −0.138	0.561
**Near work time**	0.033	0.014	0.018, 0.006	0.018*
**Duration of breastfeeding**				
Never	0 (reference)			
<6 months	−0.069	0.142	−0.348, 0.211	0.629
>6 months	0.138	0.127	−0.112, 0.388	0.278

SE, standard error; CI, confidence interval; *P < 0.05; **P < 0.01.

## Discussion

This study found a prevalence of myopia of 53.9% in 6- to 12-year-old school children in Tianjin. Myopia has been suggested to commonly occur in primary school children in Tianjin. In central China, the Anyang Childhood Eye Study showed that the prevalence rates of myopia were 3.9% and 67.3% at mean ages of 7.1 years and 12.7 years, respectively^24^. Similarly, You *et al*.^25^ reported a high prevalence of myopia (57%) in students aged 7–18 years in Beijing in 2012. Taken together, those results highlight the emergent need for efforts to control myopia in school-aged children in China.

Age was an independent and significant factor associated with the prevalence of myopia, SER and AL in this study, and previous studies reported similar results. In 2015, Guo *et al*.^26^ reported that the prevalence rate of myopia increased annually by 34% among school children in Ejina. Li *et al*.^27^ also found that the mean change in SER per year was −0.48 D, and that the mean change in AL per year was 0.24 mm.

The maternal educational level was associated with SER, because children with mothers who had university and higher educational levels had significantly more hyperopic refraction. Previous studies have reported consistent findings. Hsu *et al*.^28^ have shown that higher maternal education is a protective factor for myopia in Taipei. Sensaki *et al*.^29^ have also reported that parents with higher educational levels may tend to place a greater focus on education in their children. However, Guo *et al*. have reported conflicting results, demonstrating that a higher maternal educational level is associated with presenting visual impairment and a higher prevalence of myopia^30,31^. In this study, we deduced that mothers with university and higher educational levels might have a more comprehensive awareness of the harmful impacts of myopia and make greater efforts to cultivate good habits in their children for myopia prevention.

The parental myopic status was associated with the prevalence of myopia, SER and AL in both logistical and linear regression analyses, which was consistent with the findings in previous studies. A study in the Chaoyang District of Beijing revealed that children with two myopic parents were more likely to be myopic than children with non-myopic parents (OR = 3.10, 95% CI: 2.49–3.86)^32^. In Singapore, Saw *et al*. found that parents’ myopic histories were associated with a more negative SER and a longer AL in 1453 children aged 7–9 years^33^. Specifically, our finding provided additional evidence for the relationship between children with one myopic parent and the SER but not the prevalence rate of myopia. We deduce that the influence of one myopic parent on the SER might be insufficient to achieve a sufficient level of myopic refraction. Previous studies revealed that a parental history of myopia could better represent the shared genetic effects on myopia. However, shared environments may play an important role in myopia^34,35^. Thus, further studies are needed to explore the interaction between environmental factors and genes and provide more clues to confirm the effects of environmental factors on myopia.

Breastfeeding was associated with a decreased risk of myopia in our sample, suggesting that children who were breastfed were less likely to have myopia. An earlier cross-sectional study of 797 children aged 10–12 years in Singapore suggested that the myopia risk in breastfed children was 42% lower than that in non-breastfed children (adjusted OR = 0.58; 95% CI: 0.39, 0.84)^17^. In contrast, Rudnicka *et al*.^18^ provided evidence that no significant association existed between infant feeding and visual outcomes in a European setting after adjusting for age. Possible differences between Rudnicka *et al*.’s study and our study include the use of different study designs, varying participants ages and ethnicities, the use of distinct definitions and diagnostic criteria for refractive errors, and adjustment for different confounders in the model. Furthermore, we examined the association between breastfeeding and the mean SER and found that breastfeeding for less than 6 months was positively associated with increased hyperopia. Chong *et al*.^17^ found that the mean SER of breastfed children (−1.6 D) in Singapore was more hyperopic than that of children who were not breastfed (−2.1 D) (*P* = 0.001). Similarly, Sham *et al*.^20^ reported that the breastfeeding status was independently associated with the SER, and that the mean SER was more hyperopic for breastfed children than for those who were not breastfed after adjustment. In contrast, Ebrahim *et al*.^19^ showed no significant relationship between breastfeeding during the first 6 months of infancy and refractive errors. The ethnicity and definition of breastfeeding in our study were different from those in Ebrahim *et al*.’s study; moreover, their study did not adjust for potential confounding factors.

The results of this study also indicated that the association between breastfeeding and myopic refraction could exist in the childhood. Previous studies showed that children who were breastfed had better vision in infancy and early childhood than those who were formula fed^16,36^, and a study in Singaporean children aged 10–12 years also confirmed this association in later childhood^17^. However, breastfeeding for more than 6 months was not significantly associated with the mean SER but was significantly associated with the prevalence of myopia. This finding might be attributed to the association between breastfeeding during the first 6 months and hyperopic SER, which was likely more significant. However, this finding must be interpreted with caution, because our sample may not have had sufficient power to determine the relationship. Additional studies with a larger sample size are needed to confirm this result. However, after the first 6 months of infancy, adequate nutritious foods are recommended to complement the milk intake, which may impact the relationship between breastfeeding and SER.

Human milk may provide polyunsaturated fatty acids, essential vitamins and bioactive components to babies^37^. Increasing evidence shows that breastfed infants have better vision and neurodevelopmental outcomes^38–40^. The fatty acids in human milk usually contain approximately 0.5–0.8% arachidonic acid (ARA) and 0.1–0.4% docosahexaenoic acid (DHA)^19^. Infants should reportedly continuously intake DHA and ARA during early life, which is a critical period of infant growth and development^41^. In addition, DHA is necessary for brain and retinal growth and maturation in infants and maintains high concentrations in these tissues^40^. Long-chain polyunsaturated fatty acids are abundant in the retina, especially in the retinal photoreceptor outer segment disk membranes^42^, and influence retinal cell gene expression, cell differentiation and cell survival^43^. Similarly, animal experiments have suggested that deficient DHA levels impair neural and retinal functions^44,45^.

In this study, no significant relationship was found between the breastfeeding duration and the mean AL. This result revealed that the positive effect of breastfeeding on myopia was not mediated by AL. However, there is currently a lack of studies on the association between breastfeeding and ocular biometric parameters. In the future, we will continue to explore the relationship between breastfeeding and other ocular biometric parameters, such as corneal curvature, anterior chamber depth, and lens thickness, to determine whether the relationship between breastfeeding and myopia is mediated by other refractive parameters. Notably, the time spent near work was significantly associated with AL in the multiple linear regression model but did not have a significant relationship in the multiple regression model with the prevalence of myopia and the mean SER. Because studies have suggested that the time spent near work is significantly associated with AL^46,47^, we might infer that visual behavioural factors, such as the time spent near work, are more likely to affect the AL, whereas nutrition intake, such as breast milk, in early life may have a greater effect on the SER. Nutritional and visual behavioural factors might have different impacts on eye development. This result was also supported by the effect of DHA and ARA in breast milk on retinal and neural development compared with the effect on the AL. However, more details need to be confirmed in further studies.

Potential limitations of our study should be noted. First, data on breastfeeding and myopia-related factors were reported by parents in this questionnaire a few years after the events occurred. Although this method has been widely used in previous studies, it may lead to recall bias. Second, this study did not investigate the type of breastfeeding, such as exclusive, mostly (nonformula supplements), or partly (formula supplements). Although previous studies^19,20^ showed no significant relationship between the type of breastfeeding and refractive errors or myopia, and then type of breastfeeding was not included in our questionnaire, an exploration of whether any interaction occurs between the duration of breastfeeding and the type of breastfeeding will be useful. Therefore, details regarding the type of breastfeeding or the breastfeeding method should be collected and analysed in our future studies. Finally, since this study was cross-sectional in nature, the association between breastfeeding and myopic refraction should not be construed as causal. Longitudinal studies are needed to clarify a true cause-effect relationship.

In summary, the prevalence of myopia was 53.9% in 6–12-year-old children in Tianjin, China. The results of this study suggest that breastfeeding was independently associated with a decreased risk of myopia. Children who were breastfed for less than 6 months had a more hyperopic mean SER than children who were not breastfed. Breastfeeding during the first 6 months of infancy was associated with a more hyperopic SER. There was no significant relationship between breastfeeding and AL. Based on this finding, we conclude that breastfeeding was associated with myopic refraction and was not related to AL, and this association could exist in childhood.

## Methods

### Participants

A cross-sectional study was conducted to explore the association between breastfeeding and myopic refraction among primary school-aged children in Tianjin, China. A three-stage, stratified, cluster sampling method was used for the selection of participants. First, two districts were randomly selected from the 6 main urban districts in Tianjin. Second, one primary school was randomly chosen from each district. Finally, one class was randomly selected from each grade within the selected schools. All children in the selected classes were invited to participate in this study voluntarily except for children with strabismus, amblyopia, an eye injury or other eye-related disorders. After explaining the study design to the parents and children, written informed consent was obtained from at least one parent or legal guardian of each child. In total, 600 children were invited to join the study, and 531 children participated (88.5% response rate). Among them, 4 children who exceeded the age limit were excluded from the study. As a result, the final sample in the present study included 527 children aged 6–12 years (285 boys and 242 girls). This study was conducted between November 2016 and June 2017. The study protocol was approved by the Institutional Review Board of Tianjin Medical University and adhered to the tenets of the Declaration of Helsinki.

### Ocular examinations

All participants completed a detailed ocular examination, including intraocular pressure, slit-lamp examination of the anterior and posterior segments of the eye, cycloplegic autorefraction and AL. The intraocular pressure and slit-lamp examination were performed initially to rule out contraindications for cycloplegic agents in each child. Then, two drops of 0.5% tropicamide and 0.5% phenylephrine mixed eye drops were placed into each eye at a 5-minutes interval. Approximately 25 minutes after the second drop was administered, refraction measurements were performed with an auto-kerato-refractor (Canon Autorefractor RK-F1, Tokyo, Japan). The AL was measured by the Lenstar LS 900 biometer (Haag-Streit AG, 3098 Koeniz, Switzerland). All ocular measurements were performed three times in each eye except for the slit-lamp examination, and the means of the measurements were calculated for the statistical analysis. All examinations were performed by board-certified ophthalmologists and certified orthoptists and were completed within one hour.

### Definition of myopia

The spherical equivalent of the refractive error was calculated as the spherical value plus half of the cylindrical (astigmatic) value. Myopia was defined as a SER less than −0.5 dioptres (D). The SER values of the two eyes were significantly highly correlated (*r* = 0.873, *P* < 0.001); therefore, only the right eye of each individual was used for statistical analysis.

### Questionnaire

A questionnaire was developed to analyse possible risk factors associated with myopia based on a thorough literature review. The main variables were as follows:

The socio-demographic variables included age (calculated from the date of birth to the survey date), gender (boys/girls), and household monthly income (<4000 RMB/4000–8000 RMB/8000–10,000 RMB/10,000–15,000 RMB/>15,000 RMB).

The parental characteristics included the maternal educational level (senior high school and lower/college/university/Master’s and higher), parental myopic status (none/one/both), maternal age, maternal drinking history (yes/no), maternal smoking history (yes/no) and passive smoking history (yes/no).

The children’ birth characteristics included the child’s birth weight, birth height, mode of delivery (vaginal delivery/caesarean delivery) and gestational weeks (postmature delivery/term delivery/premature delivery).

Based on previous studies^48,49^, the amount of time spent in outdoor and near work activities are associated with myopia in children. Therefore, the outdoor and near work times were collected by the questionnaire. The children’s outdoor time was evaluated by asking how many hours the child spent in total outdoor activities per day. The total outdoor activity was defined as the sum of outdoor leisure and outdoor sporting activities. Their near work time was assessed by asking how many hours the child spent in writing, paper-based reading and using electronics per day. The near work time was defined as the total time spent writing, reading and using electronics. The above information was obtained by inquiring about the average time spent on such activities during weekdays and weekends separately. The average number of outdoor activity and near work hours per day was calculated using the formula: [(hours spent on weekday) × 5 + (hours spent on weekend day) × 2]/7.

Breastfeeding was assessed by asking whether the child was breastfed (yes/no) and the duration of breastfeeding (never/<6 months/6–12 months/>12 months).

The questionnaire was evaluated by experts in this field, and some items were revised based on their feedback. Then, twenty parents and their children were asked to participate in our pilot study. According to their suggestions, we revised the questionnaire to guarantee that the contents were suitable for the study population. The questionnaire was completed by the parents of the participants during the ocular examination. The research assistants immediately checked the questionnaire data to ensure that the contents were reasonable.

### Statistical analysis

Statistical analysis was conducted using a commercial statistical programme (SPSS for Windows, version 21.0; IBM-SPSS, Chicago, IL, USA). The Forest plot was drawn using Microsoft Excel, and the data are presented as a percentage or as the mean ± standard deviation (SD). Associations between the myopia prevalence or the SER and breastfeeding or related risk factors were identified using Chi-square tests for categorical variables and t-tests for quantitative variables. P-P plots and the Kolmogorov-Smirnov test were performed to check the distribution of our data. A multiple logistical regression model was used to determine associations between the myopia prevalence and breastfeeding after adjusting for possible confounders, including age, gender, birth height, birth weight, maternal educational level, parental myopia, monthly household income, maternal age, mode of delivery, gestational weeks, maternal smoking history during pregnancy, maternal passive smoking history during pregnancy, maternal drinking history during pregnancy, outdoor time and near work time. In addition, the SER and AL were modelled using multiple linear regression with adjustments for the same confounding factors. Odds ratios (ORs) and 95% confidence intervals (CIs) were calculated. All statistical tests were two-sided, and all *P*-values < 0.05 were considered significant.

### Data availability

The datasets generated and analysed during this study are not publicly available to protect the privacy of the participants. The data are available from the Department of Maternal, Child and Adolescent Health at the School of Public Health of Tianjin Medical University and can be obtained from the corresponding author (email: zhangxinty06@163.com) upon reasonable request.
